# DNA Methylation of Patatin-Like Phospholipase Domain-Containing Protein 6 Gene Contributes to the Risk of Intracranial Aneurysm in Males

**DOI:** 10.3389/fnagi.2022.885680

**Published:** 2022-07-11

**Authors:** Shengjun Zhou, Junjun Zhang, Chenhui Zhou, Fanyong Gong, Xueli Zhu, Xingqiang Pan, Jie Sun, Xiang Gao, Yi Huang

**Affiliations:** ^1^Department of Neurosurgery, Ningbo First Hospital, Ningbo, China; ^2^Key Laboratory of Precision Medicine for Atherosclerotic Diseases of Zhejiang Province, Ningbo, China; ^3^Department of Ultrasound, Ningbo First Hospital, Ningbo, China; ^4^Ningbo Center for Disease Control and Prevention, Ningbo, China; ^5^Medical Research Center, Ningbo First Hospital, Ningbo, China

**Keywords:** PNPLA6, DNA methylation, mRNA expression, age, intracranial aneurysms

## Abstract

**Objective**: This study is aimed to investigate the contribution of patatin-like phospholipase domain-containing protein 6 (*PNPLA6*) DNA methylation to the risk of intracranial aneurysm (IA) in the Han Chinese population.

**Methods**: A total of 96 age- and sex-matched participants were recruited to evaluate *PNPLA6* methylation *via* bisulfite pyrosequencing. The *PNPLA6* mRNA expression in the plasma was determined using real-time quantitative reverse transcription-polymerase chain reaction. Human primary artery smooth muscle cells (HPCASMC) were used for the in vitro function study.

**Results**: *PNPLA6* methylation was significantly higher in patients with IA than in healthy controls (*p* < 0.01). Sex group analysis showed that this correlation appeared in the male group (*p* < 0.01) but not in the female group (*p* > 0.05). *PNPLA6* methylation was significantly associated with age in all participants (*r* = 0.306, *p* = 0.003) and in the control group (*r* = 0.377, *p* = 0.008) but not in the IA group (*r* = 0.127, *p* = 0.402). Furthermore, the *PNPLA6* mRNA expression significantly decreased in patients with IA than that in the controls (*p* = 0.016). *PNPLA6* expression was significantly inversely correlated with elevated DNA methylation in participants (r = −0.825, *p* < 0.0001). In addition, *PNPLA6* transcription was significantly enhanced following treatment with 5-aza-2’-deoxycytidine methylation inhibitor in HPCASMC.The receiver operating characteristic analyses of curves showed that the* PNPLA6* mean methylation [area under the curve (AUC) = 0.74, *p* < 0.001] and mRNA expression (AUC = 0.86, *p* < 0.001) could have a diagnostic value for patients with IA.

**Conclusion**: Although future functional experiments are required to test our hypothesis, our study demonstrated that* PNPLA6* methylation and mRNA expression were significantly associated with the risk of IA; thus, they show potential for use in the early diagnosis of IA.

## Introduction

Intracranial aneurysm (IA) is a common cerebrovascular disease with an extremely high mortality (Lu et al., 2021). Its incidence is greater than 7% in the Chinese population aged >35 years old (Li et al., 2013). IA is a complex disease with genetic and environmental risk factors (Bakker et al., 2020; Lu et al., 2021). Tobacco and alcohol consumption, high-fat diet, age, sex, and other factors can increase the risk of IA by affecting the expression of related genes (Bakker et al., 2020; Wang et al., 2021). However, the mechanisms underlying IA pathogenesis are not yet fully understood.

DNA methylation often occurs in cytosine-phosphate-guanine (CpG) dinucleotide sequences in the mammalian genome (Moore et al., 2013). Its levels can be influenced by external factors, which can alter DNA stability, as well as its ability to interact with proteins (Zocher et al., 2021). It can regulate the expression of numerous genes, and aberrant gene methylation plays a vital role in the development of multiple diseases (Deng et al., 2019). In addition, DNA methylation and the binding of its effector proteins to methylated DNA are essential for vascular function and development (Rao et al., 2011). DNA methylation may also participate in the development of IA by regulating the expression of genes involved in inflammatory reactions, cell function, and cell signal transduction (Yu et al., 2017).

Patatin-like phospholipase domain-containing protein 6 (*PNPLA6*) is a phospholipase that deacetylates intracellular phosphatidylcholine to produce glycerophosphatidylcholine (Richardson et al., 2013). *PNPLA6* is located on human chromosome 19p13.2, which contains 37 exons and multiple mutation sites (Richardson et al., 2020). *PNPLA6* mutations are associated with many diseases (Sen et al., 2020; Wu et al., 2021) and are involved in several disorders in adult organisms and embryos (Emekli et al., 2021; Suchowersky et al., 2021). The content of the *PNPLA6* in the brain plays an important role in the balance of brain function. Loss of *PNPLA6* activity leads to abnormally elevated levels of phosphatidylcholine in the brain and damages the secretory pathway in neurons (Pamies et al., 2014b). *PNPLA6* has also been associated with chorioretinal dystrophy (Dogan et al., 2021), Parkinson’s syndrome (Sen et al., 2020), and nerve lesions (Richardson et al., 2020). *PNPLA6* likely participates in the development of neural and vascular systems in living organisms (Moser et al., 2004; Chang and Wu, 2010). Silencing the expression of *PNPLA6* causes a series of changes in functional pathways, which eventually leads to lesions in cerebrovascular system (Pamies et al., 2014a, b). IA is a cerebrovascular disease in which the walls of cerebral arteries are abnormally prominent. However, the investigation into the association between *PNPLA6* and pathological changes in arterial vessels is lacking.

In this study, we hypothesized that *PNPLA6* DNA methylation contributes to IA risk. We aimed to test the association between *PNPLA6* DNA methylation and IA in Han Chinese individuals. We also investigated the relationships among *PNPLA6* mRNA, DNA methylation, and age in homogeneous samples.

## Materials and Methods

### Sample Collection

A total of 96 age- and sex-matched individuals were recruited from the Ningbo First Hospital for the case-control study. The participants’ clinical data including age, triglycerides (TG), total cholesterol (TC), high-density lipoprotein (HDL), and low-density lipoprotein (LDL) were reported in previous studies (Wang et al., 2021). The case group was diagnosed using cerebral angiography or magnetic resonance imaging. The control group was composed of healthy subjects. Those with cardiovascular and cerebrovascular, severe liver and kidney, and other diseases were excluded. All study protocols were approved by the Ethics Committee of Ningbo First Hospital. Peripheral blood was collected from participants and coagulated at 4°C and 3,000 rpm for 15 min. The upper plasma and peripheral blood mononuclear cells were carefully aspirated for subsequent experiments.

### Pyrosequencing Assay

An automatic nucleic acid extractor (Lab-Aid 820, Xiamen, China) was used to extract DNA from peripheral blood mononuclear cells. The DNA was subjected to quality control using a NanoDrop 2000 spectrophotometer (Thermo Fisher Scientific Inc., MA, USA). Bisulfite transformation was performed using an Epi Tech DNA bisulfite kit (Qiagen, Hilden, Germany). DNA methylation levels were measured using a PyroMark Q96 ID System (Qiagen). Five CpG dinucleotides on the fragment (GRCh37/hg19, Chr19: 7, 615, 203–7, 615, 727) with *PNPLA6* were chosen to measure methylation levels. Polymerase chain reaction (PCR) amplification primers were designed using the PyroMark Assay Design software v2.0.1.15 (Qiagen). The sequences of the PCR primers were as follows: forward primer, 5’-Biotin-GGATTTGGGGGTGGTTAGA-3’; reverse primer, 5’- TACTCCCCCACCAACTCCTTCT-3’; and sequencing primer, 5’-ACCAACTCCTTCTTAC-3’.

### Quantitative Real-Time (qRT)-PCR

Among the included samples, 18 IA patients (nine males and nine females) and 18 sex-age-matched controls (nine males and nine females) were selected for RNA expression detection. Total RNA was isolated from plasma using TRIzol reagent (Invitrogen, CA, USA) and then reverse transcribed into cDNA using a high-capacity cDNA reverse transcription kit (Applied Biosystems, CA, USA). qRT-PCR amplification was performed on a LightCycler 480 system (Roche, Mannheim, Germany) by using an SYBR Green Master Mix kit (TaKaRa, Dalian, China). The transcription of *PNPLA6* was normalized to that of *ACTB*. The primer sequences for *PNPLA6* (Zhong et al., 2018) and *ACTB* (Cheng et al., 2022) were as follows: *PNPLA6* (forward) 5’-CCAAGAGTTCCGGCTGTCA-3’, (reverse) 5’-CACAATGAGGATGCAGTCGG-3’; ACTB (forward) 5’-AGCACAGAGCCTCGCCTT-3’, (reverse) 5’-CATCATCCATGGTGAGCTGG-3’.

### Cell Culture and 5-Aza-2’-Deoxycytidine Treatment

Human primary artery smooth muscle cells (HPCASMC; http://www.atcc.org/Products/All/PCS-100-021.aspx) were used for the in vitro studies. Cells were cultured at a density of 1 × 10^6^ cells/well in 6-well plates using Dulbecco’s modified eagle’s medium (DMEM) with 10% fetal bovine serum (FBS) and penicillin/streptomycin (Invitrogen, MA, USA) at 37°C for 24 h. The medium was changed every 6–8 h. 5-aza-2’-deoxycytidine (AZA) was used to examine the potential regulatory role of DNA methylation in *PNPLA6* gene transcription. Cells were treated with three different concentrations of AZA (0.5, 1.0, and 2.0 μM), and RNA was collected three days later for gene expression assays.

### Statistical Analyses

Statistical and figure analyses were performed using GraphPad Prism version 8.0 (La Jolla, CA, USA). The DNA methylation levels between the two groups were compared using paired statistical tests and presented as violin plots. Power and sample size calculation software (Nashville, TN, USA) was used for the power analysis. Correlations between mRNA expression, DNA methylation, age, TG, TC, HDL, and LDL were analyzed using Pearson’s correlation test. A receiver operating characteristic (ROC) curve was used to evaluate the sensitivity of *PNPLA6* methylation in IA diagnosis. A two-sided *p* < 0.05 was considered significant.

## Results

**Figure 1:**
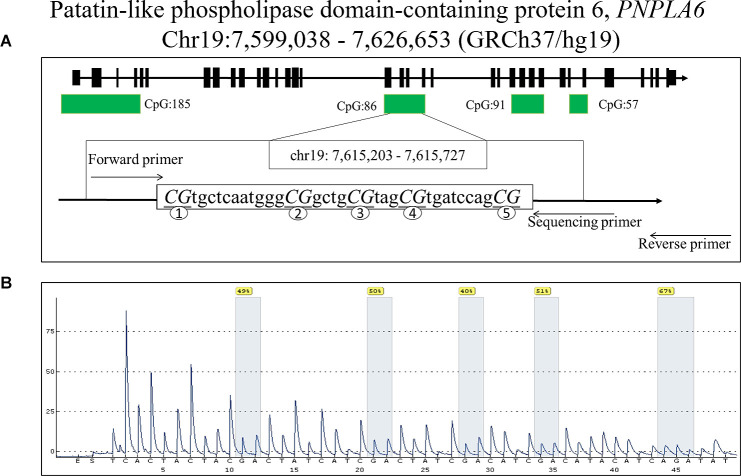
The locations and analysis of the five CpGs in *PNPLA6* gene. **(A)** The locations of the five CpGs in *PNPLA6* gene. **(B)** Representative sequencing analysis of five methylation sites.

**Figure 2:**
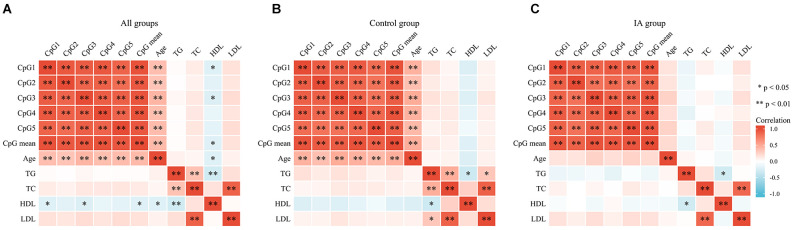
The correlations among GpGs methylation and clinical data in different subgroups. **(A)** The correlation analysis in all groups. **(B)** The correlation analysis in the control group. **(C)** The correlation analysis in IA group. The correlations among the five CpGs methylation were analyzed using Pearson’s correlation test, ^**^p < 0.01; ^*^p < 0.05.

**Figure 3:**
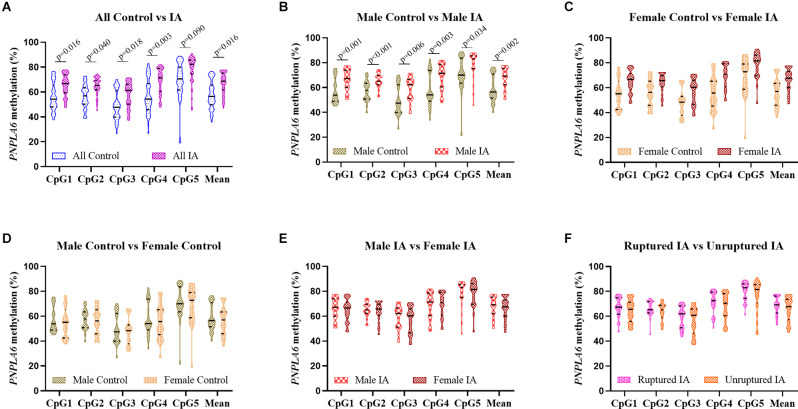
Comparison of the five GpGs methylation levels in different subgroups. **(A)** The comparison between controls and IAs in males and females. **(B)** The comparison between controls and IAs in males. **(C)** The comparison between controls and IAs in females. **(D)** The comparison between males and females in the control group. **(E)** The comparison between males and females in the IA group. **(F)** The comparison between ruptured IA and unruptured IA. Data were represented as mean ± SD.

A total of 48 subjects with IA (24 males and 24 females, mean age: 48.08 ± 5.69 years) and 48 controls (24 males and 24 females, mean age: 46.63 ± 6.04 years, *p* > 0.05) were recruited. The clinical information including TG, TC, HDL, and LDL was presented in our previous study (Wang et al., 2021) and was not statistically different between IA and control groups (*p* > 0.05). The five selected CpG dinucleotides on the fragment (GRCh37/hg19, Chr19: 7, 615, 203–7, 615, 727) with *PNPLA6* in the methylation assay are shown in Figure 1. The DNA methylation levels in the five CpG dinucleotides significantly correlated with each other in all participants (Figure 2, *p* < 0.01). There were no significant associations between *PNPLA6* methylations and clinical data such as TG, TC, HDL, and LDL (Figure 2, *p* > 0.05). *PNPLA6* methylation was significantly higher in patients with IA than in healthy controls (CpG1, *p* = 0.016, CpG2, *p* = 0.040, CpG3, *p* = 0.018, CpG4, *p* = 0.003 and mean methylation, *p* = 0.016, Figure 3A). Power analysis showed that the CpGs methylation had more than 80% power to detect the significant associations based on the nominal type I error rate of 0.01. Sex group analysis showed that this correlation only appeared in the male group (CpG1, *p* = 0.001, CpG2, *p* < 0.001, CpG3, *p* = 0.006, CpG4, *p* = 0.003, CpG5, *p* = 0.034 and mean methylation, *p* = 0.002, Figure 3B) but not in the female group (CpG1–5 and mean methylation, *p* > 0.05, Figure 3C). Subsequent sex comparison analysis showed no sex difference between the control (CpG1–5 and mean methylation, *p* > 0.05, Figure 3D) and IA groups (CpG1–5 and mean methylation, *p* > 0.05, Figure 3E). The comparison of the ruptured aneurysms revealed no differences in *PNPLA6* methylation between the ruptured IA and unruptured IA groups (CpG1–5 and mean methylation, *p* > 0.05, Figure 3F).

**Figure 4:**
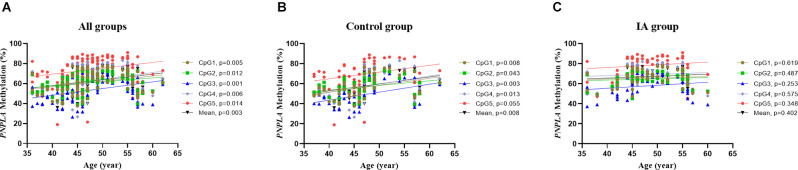
The relationship between *PNPLA6* methylations and age in different subgroups. **(A)** The relationship analysis in all participants. **(B)** The relationship analysis in the control group. **(C)** The relationship analysis in the IA group.

**Figure 5:**
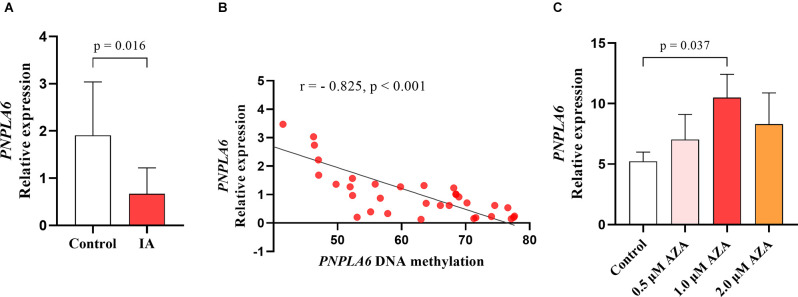
The significant association between *PNPLA6* mRNA expression and DNA methylation. **(A)** *PNPLA6* mRNA expression was much lower in IAs than in healthy controls. **(B)** The *PNPLA6* expression was significantly associated with DNA methylation in all individuals. **(C)** The changes of *PNPLA6* expression in the cell lines treated with AZA.

Correlation tests were performed to analyze the relationship between *PNPLA6* methylation and age. The results showed that *PNPLA6* methylation was significantly associated with age in all participants (mean methylation: *r* = 0.306, *p* = 0.003, Figure 4A) and the control group (mean methylation: *r* = 0.377, *p* = 0.008, Figure 4B) but not the IA group (*r* = 0.127, *p* = 0.402, Figure 4C). Furthermore, *PNPLA6* mRNA expression significantly decreased in patients with IA compared with that in the controls (*p* = 0.016, Figure 5A). Moreover, *PNPLA6* expression was significantly inversely correlated with elevated DNA methylation in participants (*r* = −0.825, *p* < 0.001, Figure 5B). In addition, the results of methylase inhibitor AZA treatment of HPCASMC showed that the *PNPLA6* gene expression in cells treated with AZA at a concentration of 1.0 μM was significantly higher than that in the control group (*p* = 0.037, Figure 5C).

**Figure 6:**
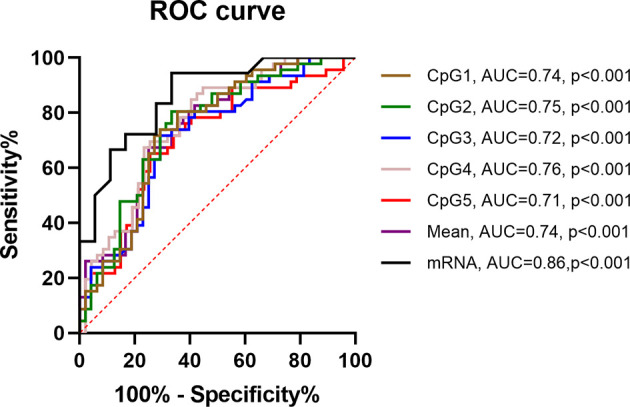
ROC curves of *PNPLA6* DNA methylation in IA patients. ROC, receiver operating characteristic. AUC, area under the curve.

ROC curves were used to evaluate the *PNPLA6* diagnostic value in patients with IA. The area under the curve (AUC) of *PNPLA6* mRNA expression was 0.86 (95% CI, 0.74–0.98, *p* < 0.001), and *PNPLA6* mean methylation was 0.74 (95% CI, 0.60–0.88; *p* < 0.001; Figure 6).

## Discussion

In the present study, we aimed to explore the association between *PNPLA6* methylation and the risk of IA. First, our results showed that plasma *PNPLA6* expression was much lower in patients with IA than in controls. Second, *PNPLA6* methylation levels were significantly higher in patients with IA than in controls, and these differences were found only in male patients. Third, *PNPLA6* methylation was inversely associated with *PNPLA6* mRNA expression in the study participants. Fourth, DNA methylation may serve an important role in the regulation of *PNPLA6* transcription in HPCASMC. Fifth, *PNPLA6* DNA methylation and mRNA expression levels had diagnostic value in patients with IA. Lastly, *PNPLA6* methylation was significantly associated with age in all participants and in the control group but not in the IA group.

The PNPLA6 protein is mainly located on the surface of the cytoplasmic endoplasmic reticulum, and concentrated in the neurons of the brain, placenta, kidney, and vascular (Richardson et al., 2013). *PNPLA6* expression is strongly associated with nervous system integrity and maintenance (Sogorb et al., 2016). *Pnpla6* silencing significantly alters the formation of the respiratory tube and nervous system (Winrow et al., 2003) and impairs vasculogenesis and placental vasculature in a mouse model (Moser et al., 2004). *PNPLA6* overexpression significantly promotes the migration and tube formation of human umbilical vein endothelial cells (HUVECs) (Li et al., 2021), and *PNPLA6* short hairpin RNA (shRNA) inhibits the migration and tube formation of HUVECs (Li et al., 2021). In the current study, the results showed that the level of *PNPLA6* expression was much lower in patients with IA than in the controls possibly because of the lower *PNPLA6* expression in patients with IA than in the controls, consequently, the risk of angiogenic lesions increases.

Studies have shown that DNA methylation influences the development of many diseases by regulating gene expression (He et al., 2022; Zhu et al., 2022). In the development of cerebrovascular disease,DNA methylation may trigger lesions by altering the expression levels of genes related to vasoconstriction or vasoproliferation, which in turn affects changes in the levels of proteins related to vascular stability (He et al., 2022). DNA methylation is closely associated with the risk of IA (Nikkola et al., 2015; Zhou et al., 2017; Shafeeque et al., 2020; Wang et al., 2021). Kim et al.’s study (Kim et al., 2022) showed that different genes with DNA methylation can be useful biomarkers for the accurate diagnosis of delayed cerebral ischemia after aneurysmal subarachnoid hemorrhage. DNA methylation participates in IA development possibly by modulating the expression of genes involved in immune and inflammatory reactions, cell signal transduction, and vascular stability (Yu et al., 2017). In other aneurysm-related diseases, Toghill et al.’s study (Toghill et al., 2018) found that *SMYD2* gene promoter methylation may be involved in the pathobiological development of abdominal aortic aneurysm by reducing *SMYD2* gene expression. In the present study, the cell experiments showed that DNA methylase inhibitor significantly upregulated *PNPLA6* transcription levels in the HPCASMC, which suggested that DNA methylation may serve an important role in the regulation of *PNPLA6* transcription. The clinical results suggested that *PNPLA6* methylation levels were significantly higher in patients with IA than in controls, and *PNPLA6* expression was inversely associated with *PNPLA6* methylation in the study participants. Thus, *PNPLA6* methylation may increase the risk of IA by regulating its mRNA expression. Moreover, ROC analyses revealed that *PNPLA6* DNA methylation and mRNA expression levels have a potential diagnostic value for IA.

Sex dichotomous effects and age are implicated in the risk factors of IA and many gene methylation rates (Vlak et al., 2011; Unnikrishnan et al., 2019; Li and Liu, 2021). The prevalence of IA and the risk of aneurysmal rupture in females are higher than those in males (Zuurbier et al., 2022). DNA methylation also shows strong sex-specific differences when individuals are exposed to harsh environments (Curtis et al., 2020). Recent studies had shown that multiple gene methylation was associated with gender differences in cardiovascular and cerebrovascular diseases (Asllanaj et al., 2020). Qin et al.’s study (Qin et al., 2019) showed that hypermethylation of ATP-binding cassette G1 gene was significantly associated with carotid intima–media thickness in males. Wang et al.’s study (Wang et al., 2016) suggested that sex modulates the interaction of *NOS1AP* promoter DNA methylation in patients with IA. Our results revealed that *PNPLA6* methylation occurred only in male patients with IA but not in females. In humans, DNA methylation levels are strongly associated with age (Horvath and Raj, 2018). The DNA methylation levels of different genes may gradually increase or decrease with age in healthy humans (Sen et al., 2016). Furthermore, DNA methylation can be used to predict chronological age (Noroozi et al., 2021). In the present study, *PNPLA6* DNA methylation levels gradually increased with age in the healthy controls but not in the patients with IA possibly because of DNA methylation disorders caused by vascular damage in patients with IA.

Our study had some limitations. First, only five GpGs on the fragment (chr19: 7, 615, 203–7, 615, 727) were selected to represent *PNPLA6*. Therefore, more CpGs analysis should be included in future studies. Second, although the subjects included in this study were sex- and age-matched, we cannot exclude those other factors including surgical treatment, medication, dietary habits, and cellular heterogeneity that may affect methylation differences. Third, although this study had great statistical power, the sample size included in this study was relatively small, more sample tests for DNA methylation and gene expression should be conducted in the future to confirm our findings. Fourth, only the DNA methylation and mRNA expression of the *PNPLA6* gene were studied in this study, and changes in protein levels would be more helpful in revealing its relationship to the pathogenesis of IA. Fifth, a candidate study was performed but a mechanistic investigation *in vitro*, in silico, or *in vivo* are needed to further verify and validate the results.

## Conclusion

Although future functional experiments are required to test our hypothesis, our findings suggest that *PNPLA6* methylation may contribute to an increased risk of IA in males by regulating its mRNA expression. Thus, *PNPLA6* methylation and mRNA expression have the potential for use in the early diagnosis of IA.

## Data Availability Statement

The raw data supporting the conclusions of this article will be made available by the authors, without undue reservation.

## Ethics Statement

The studies involving human participants and all study protocols were reviewed and approved by the Ethics Committee of Ningbo First Hospital. The patients/participants provided their written informed consent to participate in this study.

## Author Contributions

XG and JS contributed to the conception and design of the study. SZ, JZ, CZ, and FG organized the database and experiments. XZ and XP performed the statistical analysis. SZ and YH wrote the first draft of the manuscript. All authors contributed to the article and approved submitted version.

## Conflict of Interest

The authors declare that the research was conducted in the absence of any commercial or financial relationships that could be construed as a potential conflict of interest.

## Publisher’s Note

All claims expressed in this article are solely those of the authors and do not necessarily represent those of their affiliated organizations, or those of the publisher, the editors and the reviewers. Any product that may be evaluated in this article, or claim that may be made by its manufacturer, is not guaranteed or endorsed by the publisher.
